# Supplementary figures and images for: Profiling DNA break sites and transcriptional changes in response to contextual fear learning
Source: PLoS One. 2021 Jul 1;16(7):e0249691. doi: 10.1371/journal.pone.0249691 (PMC8248687; doi:10.1371/journal.pone.0249691)

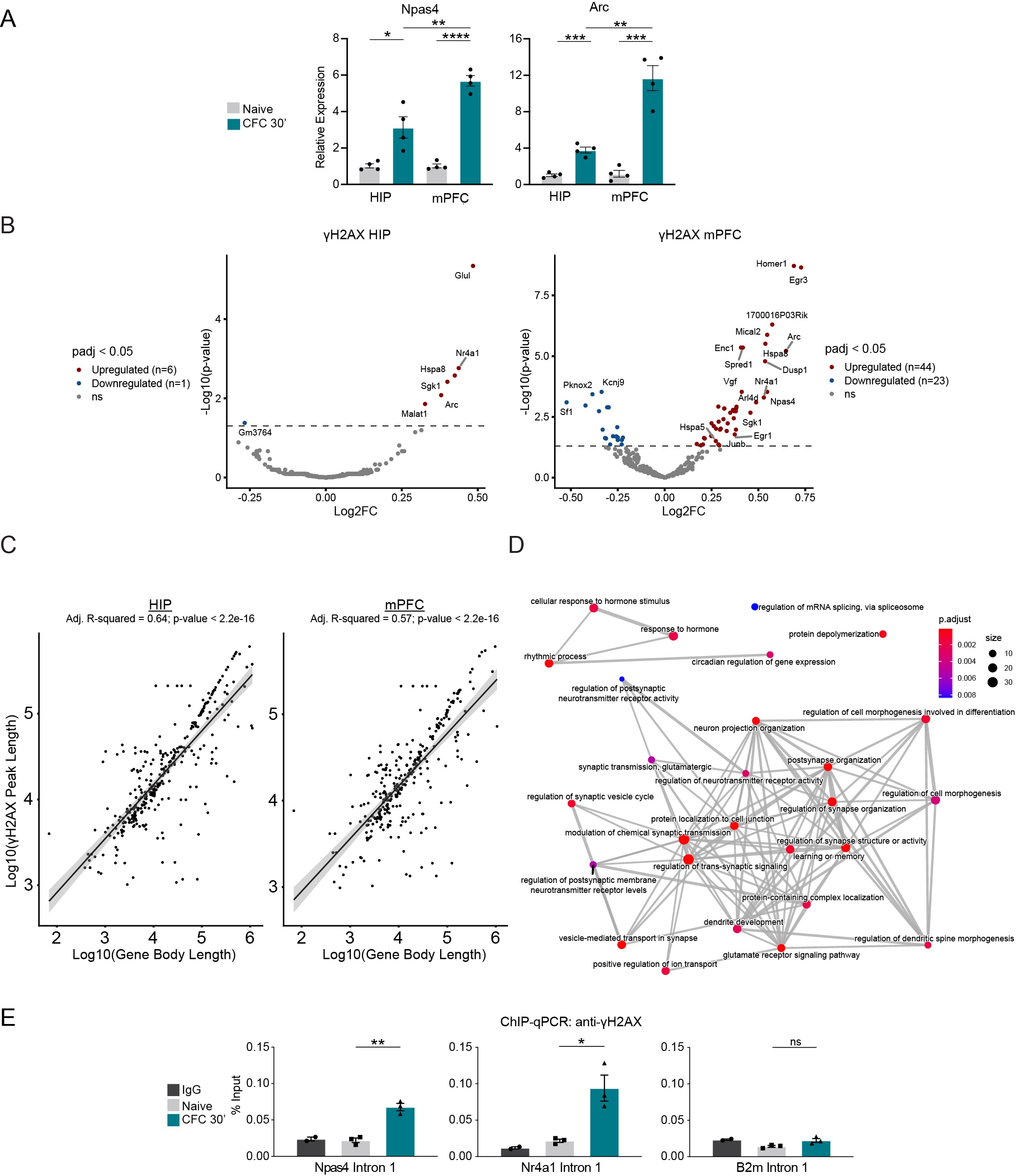

Supplement: S1 Fig — (A) qRT-PCR analysis of Npas4 and Arc mRNA expression in the hippocampus (HIP) and medial prefrontal cortex (mPFC) 30 minutes following contextual fear conditioning (CFC30’), normalized to Hprt and respective naive condition. N = 4 mice per group; two-tailed unpaired student’s t-test; * P ≤ 0.05; **P ≤ 0.01; *** P ≤ 0.001; **** P <0.0001; Mean ± SEM. (B) Volcano plots of Log2FC versus Log10(FDR) of γH2AX peaks and their corresponding genes for HIP (left) and mPFC (right). Upregulated indicates FDR < 0.05 and log2FC > 0, Downregulated indicates FDR < 0.05 and log2FC < 0, ns indicates FDR > 0.05. (C) Correlation between gene length and γH2AX peak length with linear regression. Left is HIP, right is mPFC. (D) Enrichment map of the 27 enriched biological processes for the 206 γH2AX peak-containing genes shared between HIP and mPFC in Fig 1A. Over-representation analysis with gene ontology (GO) category “Biological Process.” (E) Hippocampal γH2AX ChIP-qPCR analysis at the gene bodies of early response genes Npas4, Nr4a1, and housekeeping gene B2M. Each replicate was generated from the pooling of 3 animals’ hippocampi. N = 3; IgG N = 2; two-tailed unpaired student’s t-test; ns P > 0.05; * P ≤ 0.05; ** P ≤ 0.01; Mean ± SEM. (TIF) [file pone.0249691.s005.tif]

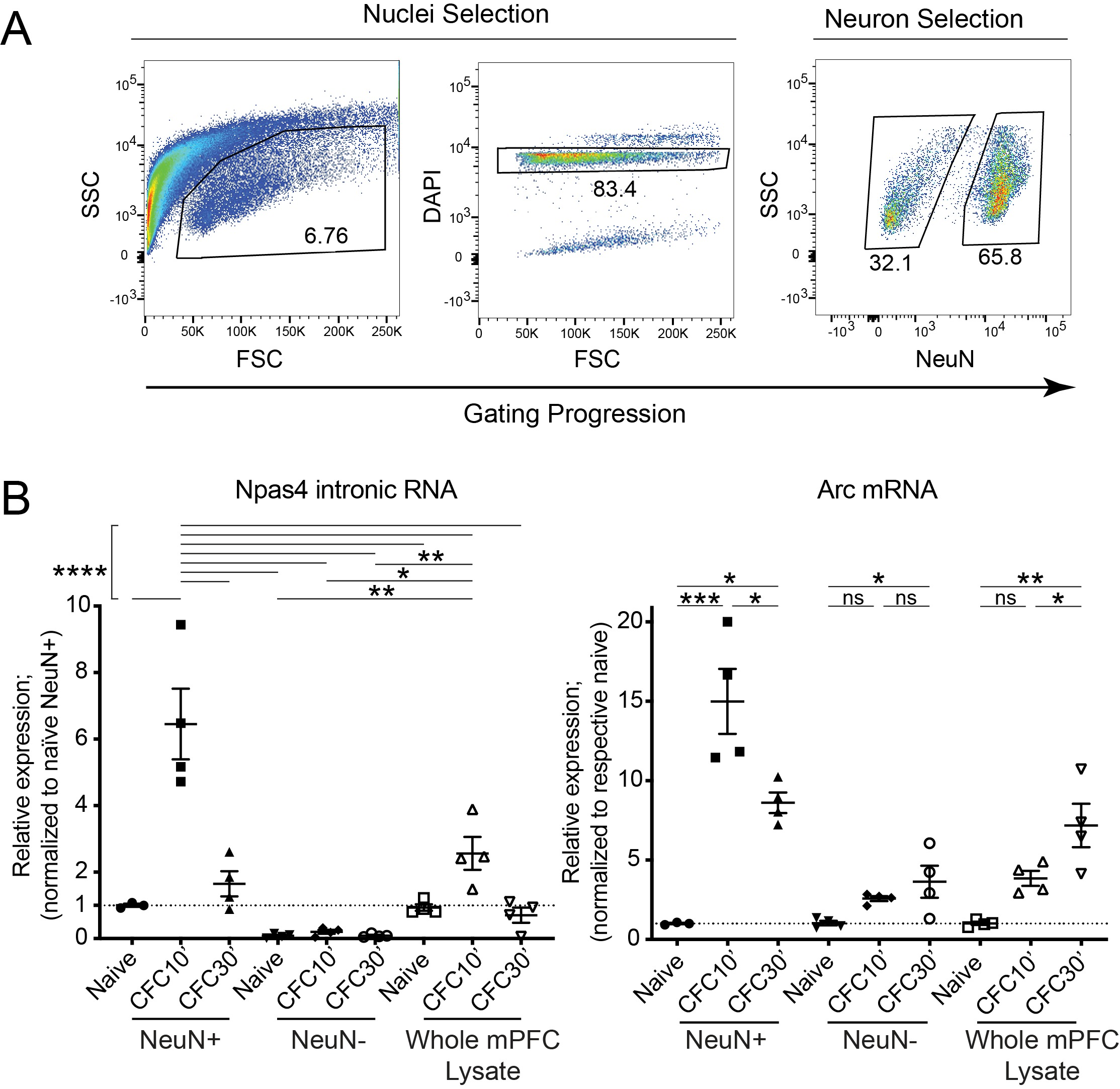

Supplement: S2 Fig — (A) Flow cytometry dot-plots representative of the gating strategy used for isolating neuronal and non-neuronal nuclei from mouse brain for RNA-Seq. Appropriately sized (FSC vs SSC), singlet nuclei (DAPI+), were gated for the presence or absence of the neuronal nuclei marker NeuN (NeuN+). (B) qRT-PCR analysis of pre-mRNA transcription from the neuronal early response gene Npas4 and mRNA for the early response gene Arc. RNA purified from FACS-isolated nuclei or whole mPFC homogenate after CFC. cDNA was primed with random hexamers and the primer used for qPCR was either intronic (Npas4) or spanned an exon-exon junction (Arc). Normalized to Hprt and relative to naive NeuN+ for Npas4 or normalized to respective naive condition for Arc. N = 3–4 mice per group; One-way ANOVA with Tukey’s multiple comparisons test; absence of an asterisk indicates P > 0.05; * P ≤ 0.05; ** P ≤ 0.01; *** P ≤ 0.001; **** P <0.0001; Mean ± SEM. (TIF) [file pone.0249691.s006.tif]

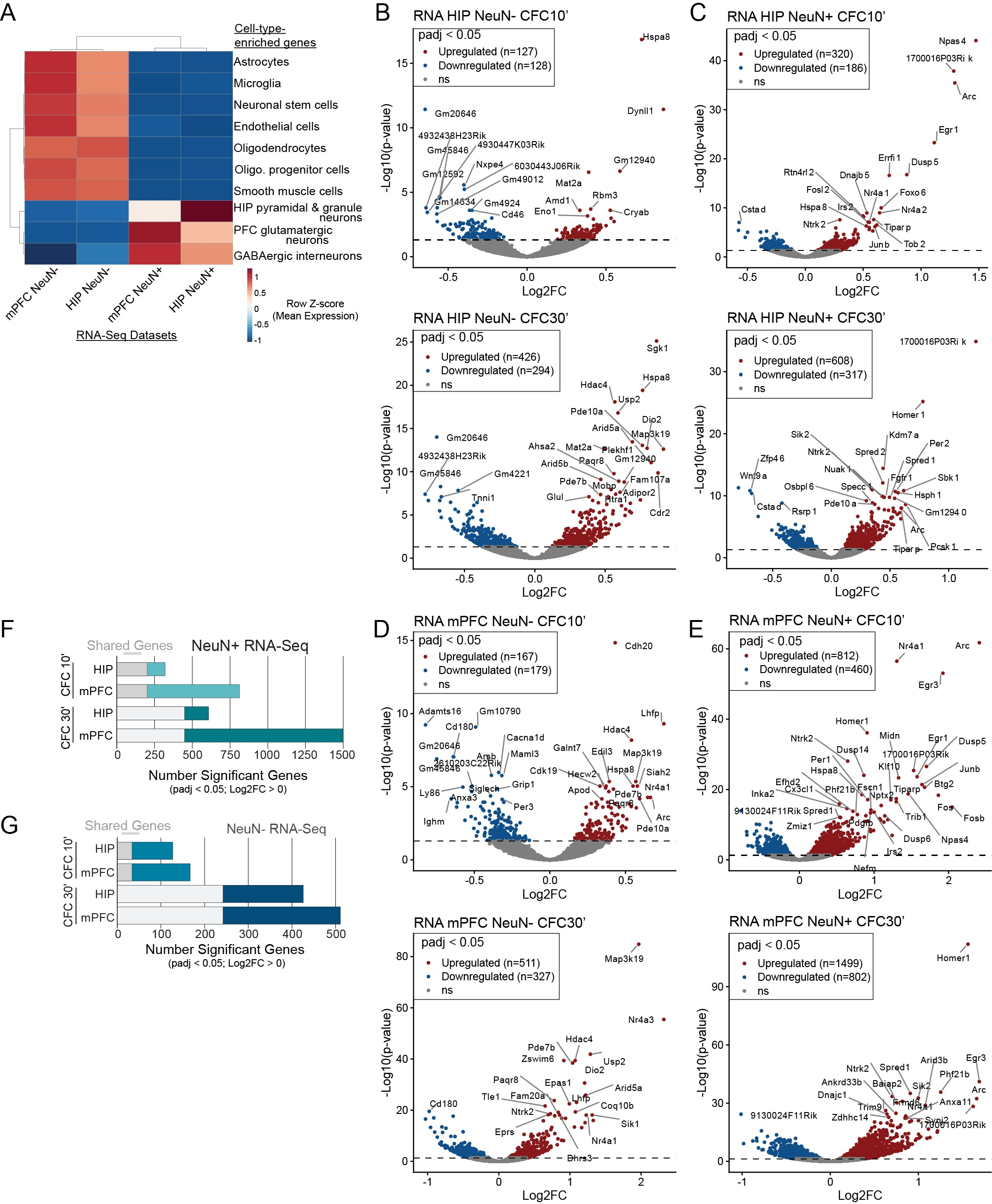

Supplement: S3 Fig — (A) Correspondence between RNA-Seq datasets and brain cell types. Marker gene sets for brain cell types was obtained from a previously published dataset [28], and the average expression of these genes was calculated (RPKM geometric mean) for the naive conditions. Z-score determined by row. (B-E) Volcano plots of Log2FC versus log10(FDR) of RNA-Seq from HIP NeuN- (A), HIP NeuN+ (B), mPFC NeuN- (C), and mPFC NeuN+ (D). Up-regulated indicates FDR < 0.05 and log2(FC) > 0, Down-regulated indicates FDR < 0.05 and log2(FC) < 0, ns indicates FDR > 0.05. (F-G) Number of upregulated genes in neuronal (F) and non-neuronal (G) nuclei 10 to 30 minutes following CFC (RNA-Seq; FDR < 0.05). Genes shared between HIP and mPFC are in grey (CFC10’) and light grey (CFC30’). (TIF) [file pone.0249691.s007.tif]

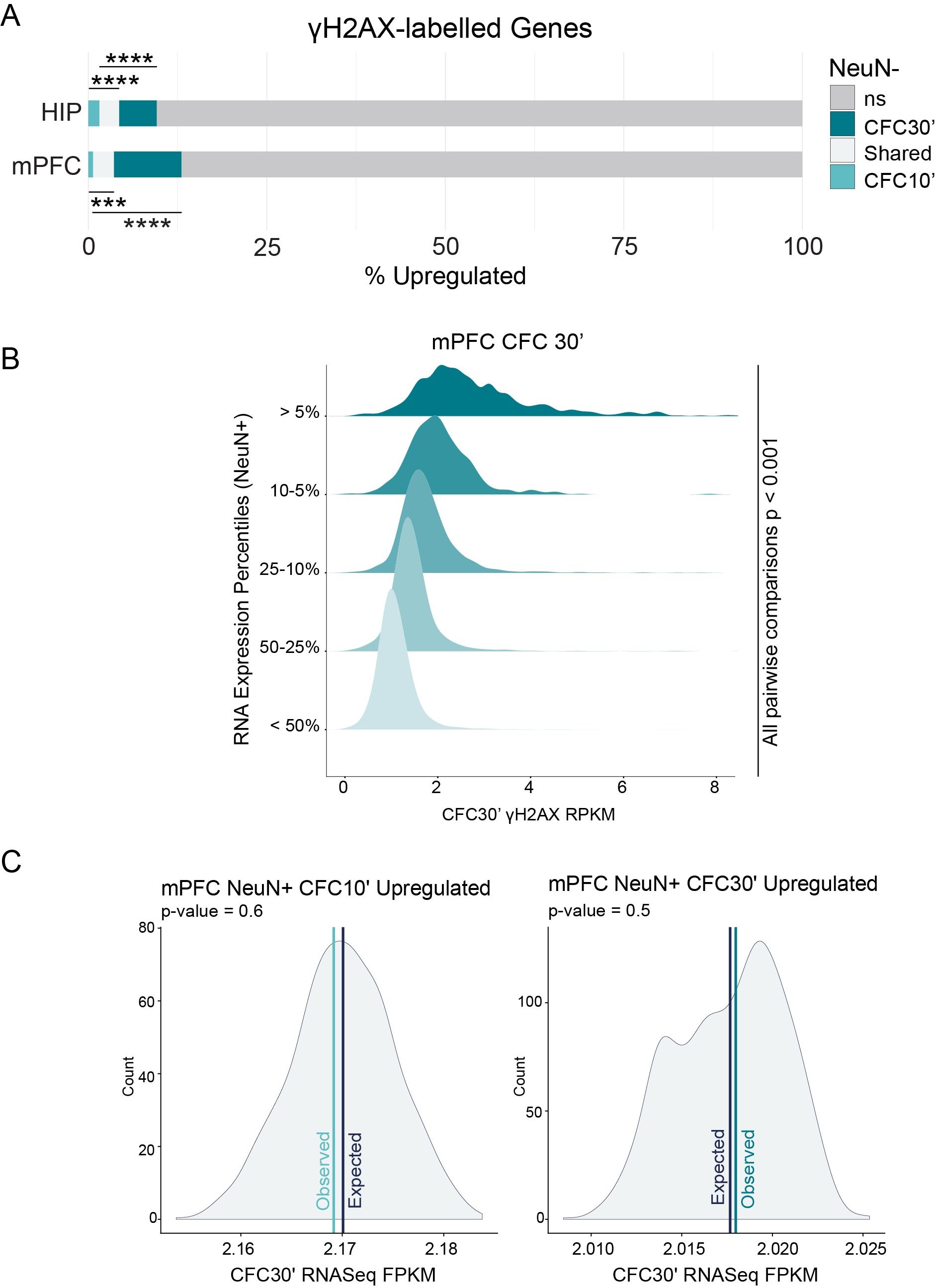

Supplement: S4 Fig — (A) Percent overlap between genes containing a γH2AX peak and those genes upregulated (padj < 0.05) in non-neuronal nuclei after CFC. Hypergeometric distribution test; *** P ≤ 0.001; **** P <0.0001. (B) DSBs increase with RNA expression level; mPFC CFC30 γH2AX ChIP-Seq intensity at gene bodies versus percentile of mPFC NeuN+ CFC30’ RNA expression. Welch’s ANOVA with Games-Howell post-hoc test. (C) Confirming equivalent RNA expression levels between the observed (upregulated) genes and expected (randomly sampled) genes for the permutation testing. Distribution of mean RNA FPKM for 1000 permutations of random sampling, binned by RNA expression level. Lines are the mean RNA FPKM. mPFC 10 minutes after CFC (left), or 30 minutes after CFC (right). (TIF) [file pone.0249691.s008.tif]

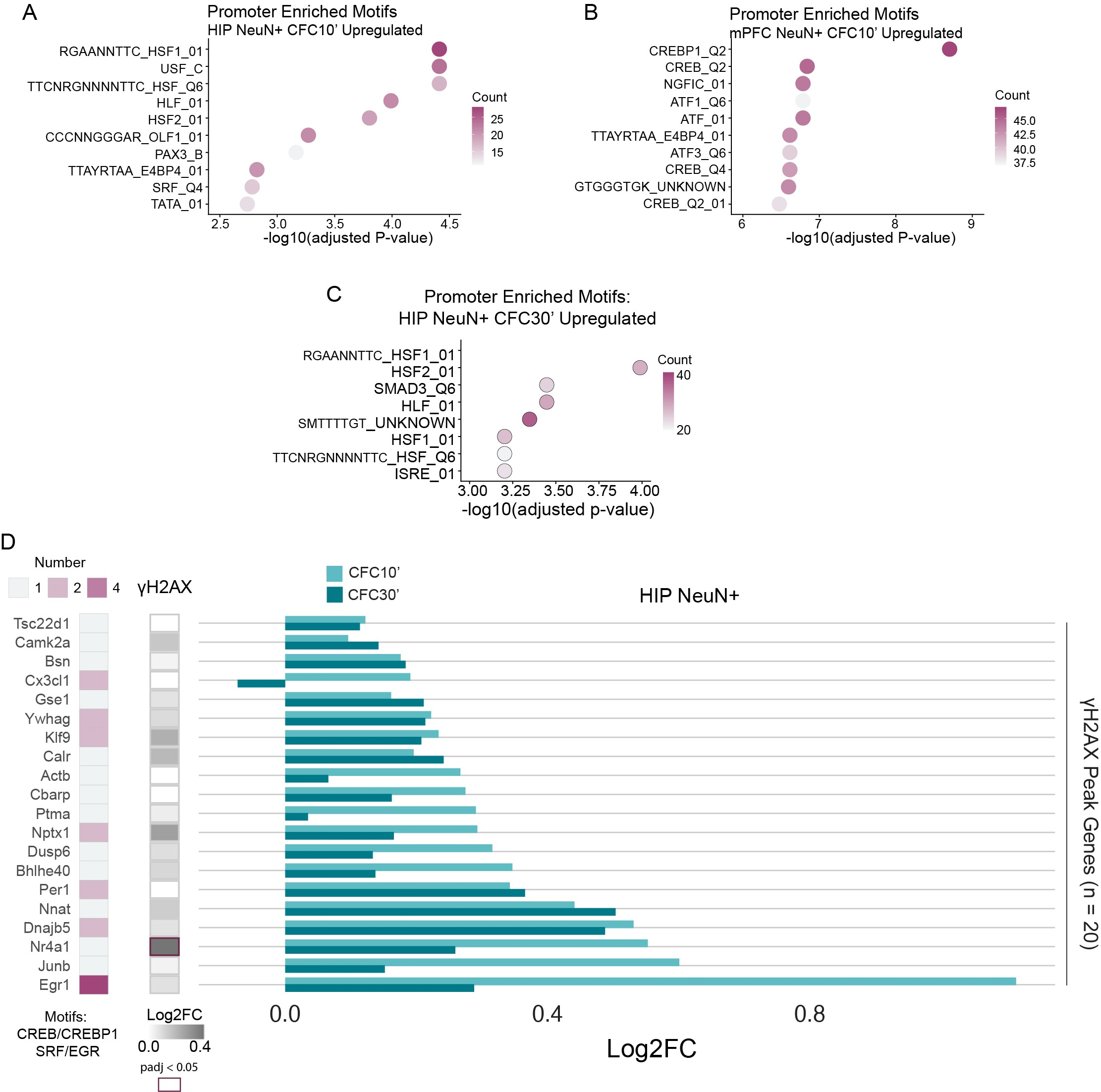

Supplement: S5 Fig — (A-B) Top 10 enriched promoter motifs for the genes upregulated in NeuN+ CFC10’ HIP (A) and NeuN+ CFC10’ mPFC (B). Using the “Transcription Factor Targets” (TFT) gene set from the molecular signatures database (MSigDB). (C) Top 8 enriched promoter motifs for the genes upregulated in neuronal nuclei from HIP 30 minutes after CFC (Log2FC >0 & FDR < 0.05). Using the “Transcription Factor Targets” (TFT) gene set from the molecular signatures database (MSigDB). (D) Motifs associated with upregulated genes in HIP NeuN+ nuclei. Left, number of the indicated motifs associated with each gene’s promoter. Center, γH2AX Log2FC, right, RNA-Seq Log2 fold change 10 or 30 minutes after CFC. Using the TFT gene sets from MSigDB for each transcription factor motif. (TIF) [file pone.0249691.s009.tif]

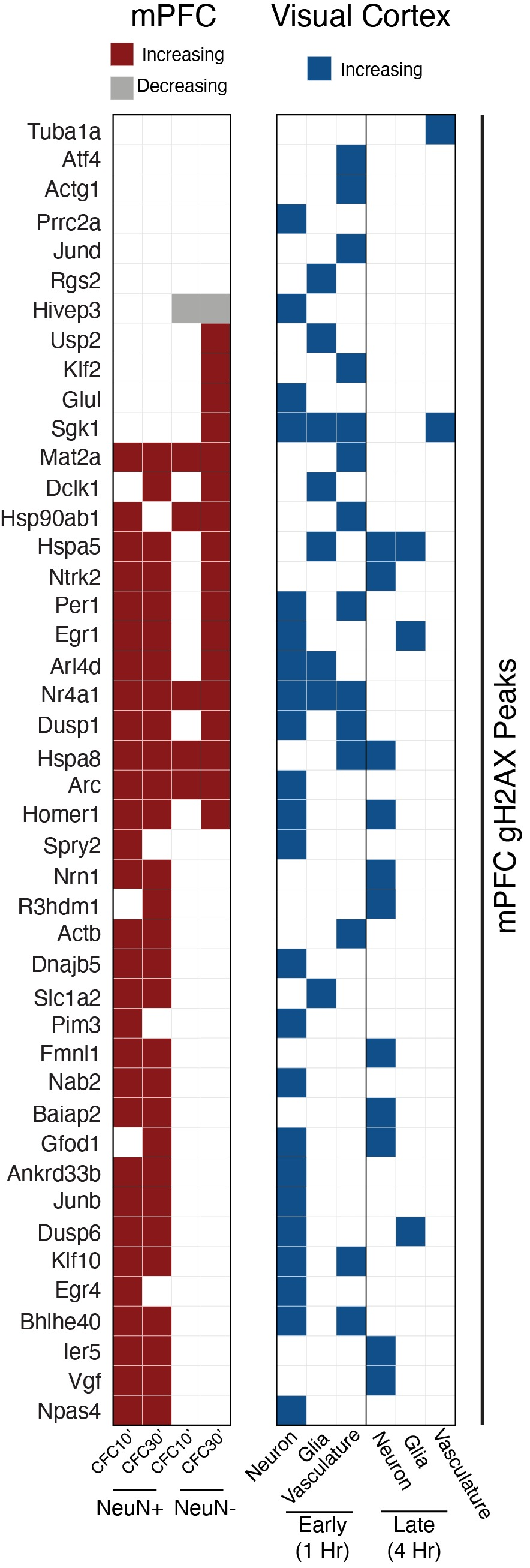

Supplement: S6 Fig — Differentially regulated genes from visual cortex single-cell RNA-Seq that also contain γH2AX peaks in mPFC (right), and their regulation in mPFC nuclear RNA-Seq (left). ‘Neuron’ encompasses excitatory and inhibitory neuron subtypes, ‘Glia’ includes all subtypes from oligodendrocytes, microglia, astrocytes, and oligodendrocyte precursor cells, while ‘Vasculature’ denotes endothelial, pericyte, and smooth muscle cell subtypes [42]. (TIF) [file pone.0249691.s010.tif]

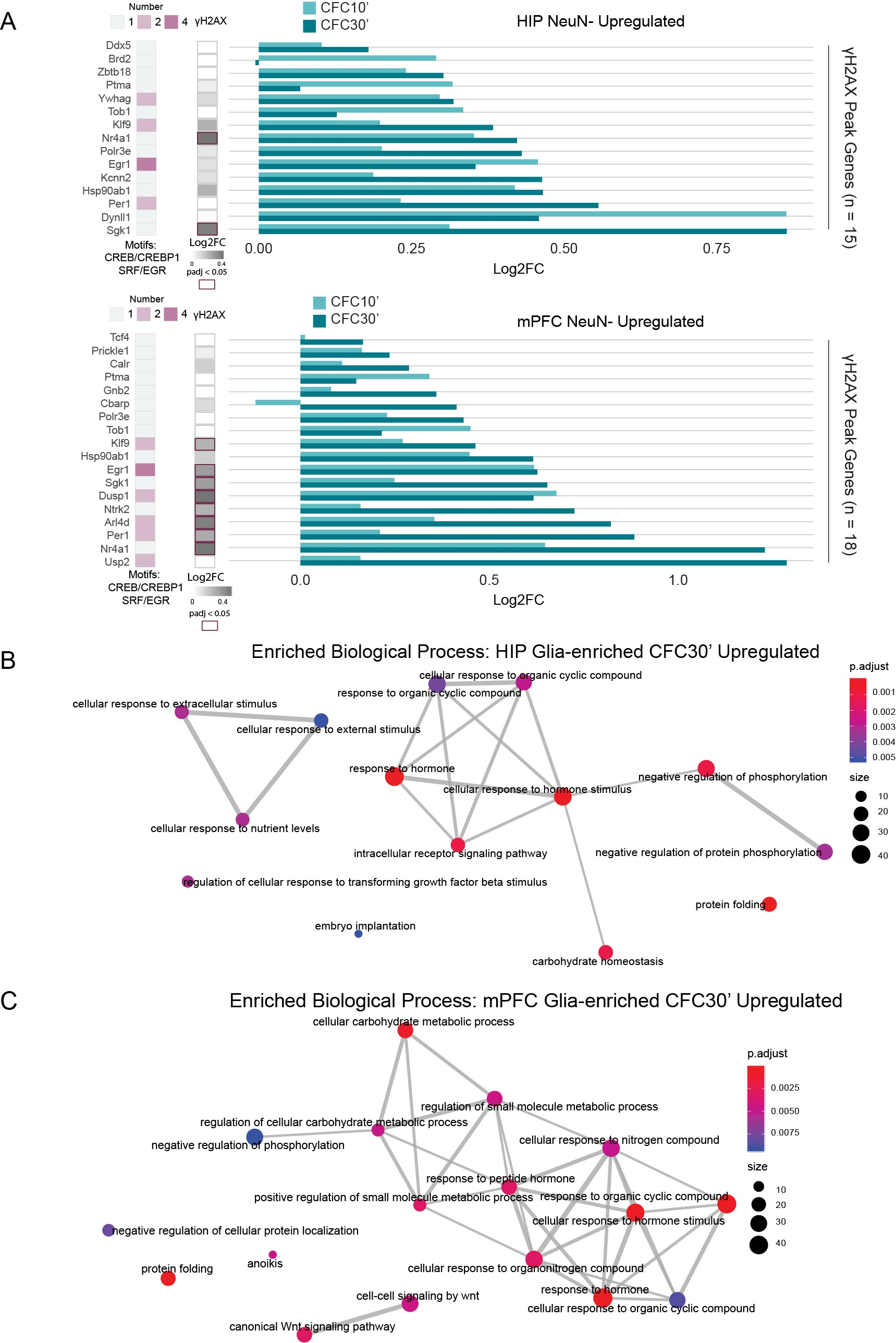

Supplement: S7 Fig — (A) Motifs associated with upregulated genes in non-neuronal nuclei from HIP (top) or mPFC (bottom). Left, number of the indicated motifs associated with each gene’s promoter. Center, γH2AX Log2FC, right, RNA-Seq Log2 fold change 10 or 30 minutes after CFC. Using the TFT gene sets from MSigDB for each transcription factor motif. (B-C) Enrichment map of the top enriched biological processes for the genes upregulated in HIP NeuN- (B) or mPFC NeuN- (C) nuclei 30 minutes after CFC. Over-representation analysis with gene ontology (GO) category “Biological Process.” (TIF) [file pone.0249691.s011.tif]

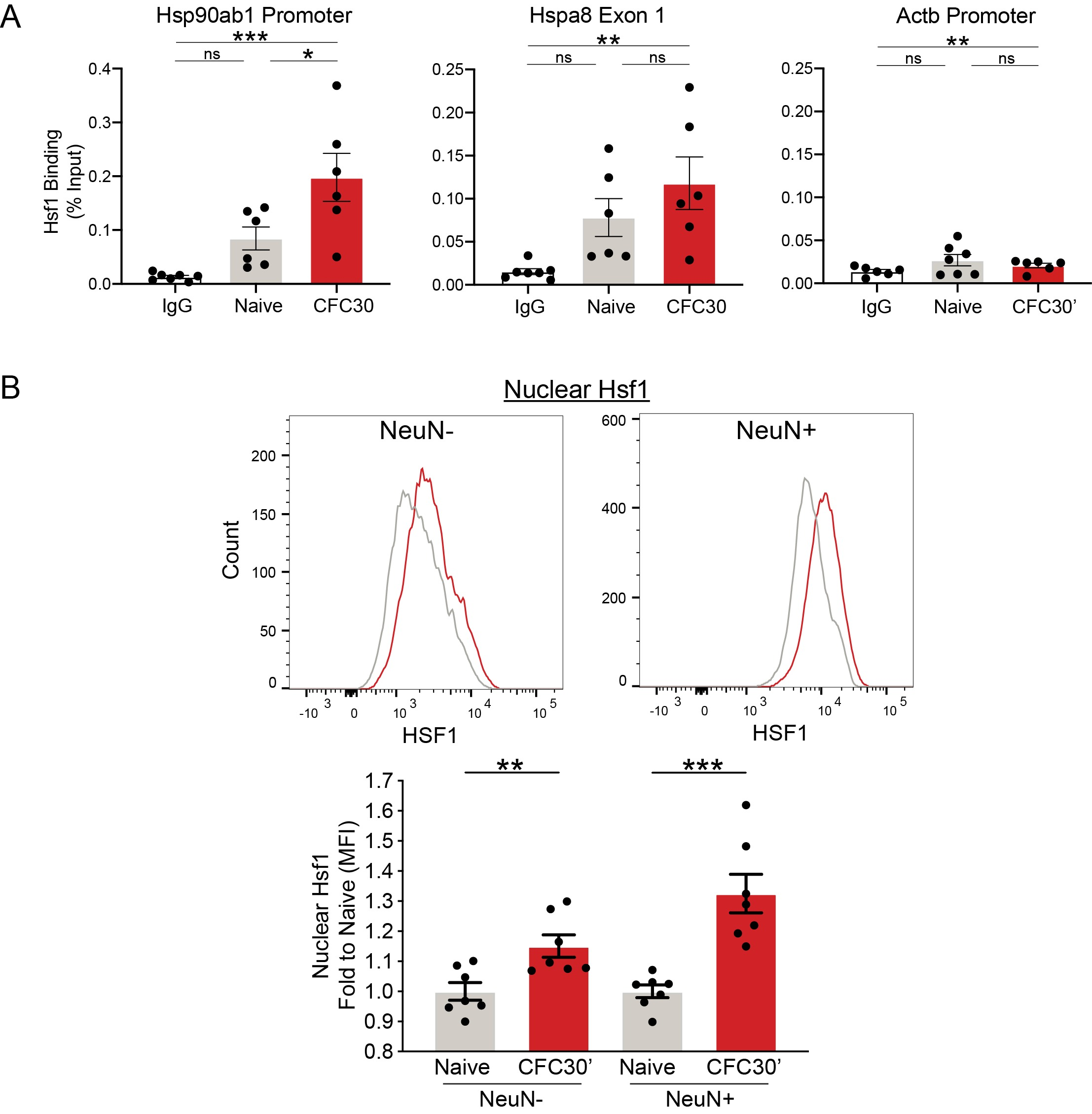

Supplement: S8 Fig — (A) HSF1 binding increases at the promoter regions of chaperones Hsp90ab1 and Hspa8. Actb, which is not a HSF1 target but has similarly increased levels of γH2AX and gene expression showed lower HSF1 binding. ChIP-qPCR of cortex 30 minutes following CFC. N = 6–7; One-way ANOVA with Tukey’s multiple comparisons test; ns P > 0.05; * P ≤ 0.05; ** P ≤ 0.01; *** P ≤ 0.001; Mean ± SEM. (B) Increased HSF1 nuclear translocation in neurons and non-neurons after CFC in cortex. Top, representative fluorescence intensity histograms after CFC (naïve, grey; CFC30, red). Bottom, intensity of nuclear HSF1 after CFC was analyzed by flow cytometry; median fluorescence intensity (MFI) normalized to respective naive condition. N = 7; two-tailed unpaired student’s t-test; ** P ≤ 0.01; *** P ≤ 0.001; Mean ± SEM. (TIF) [file pone.0249691.s012.tif]

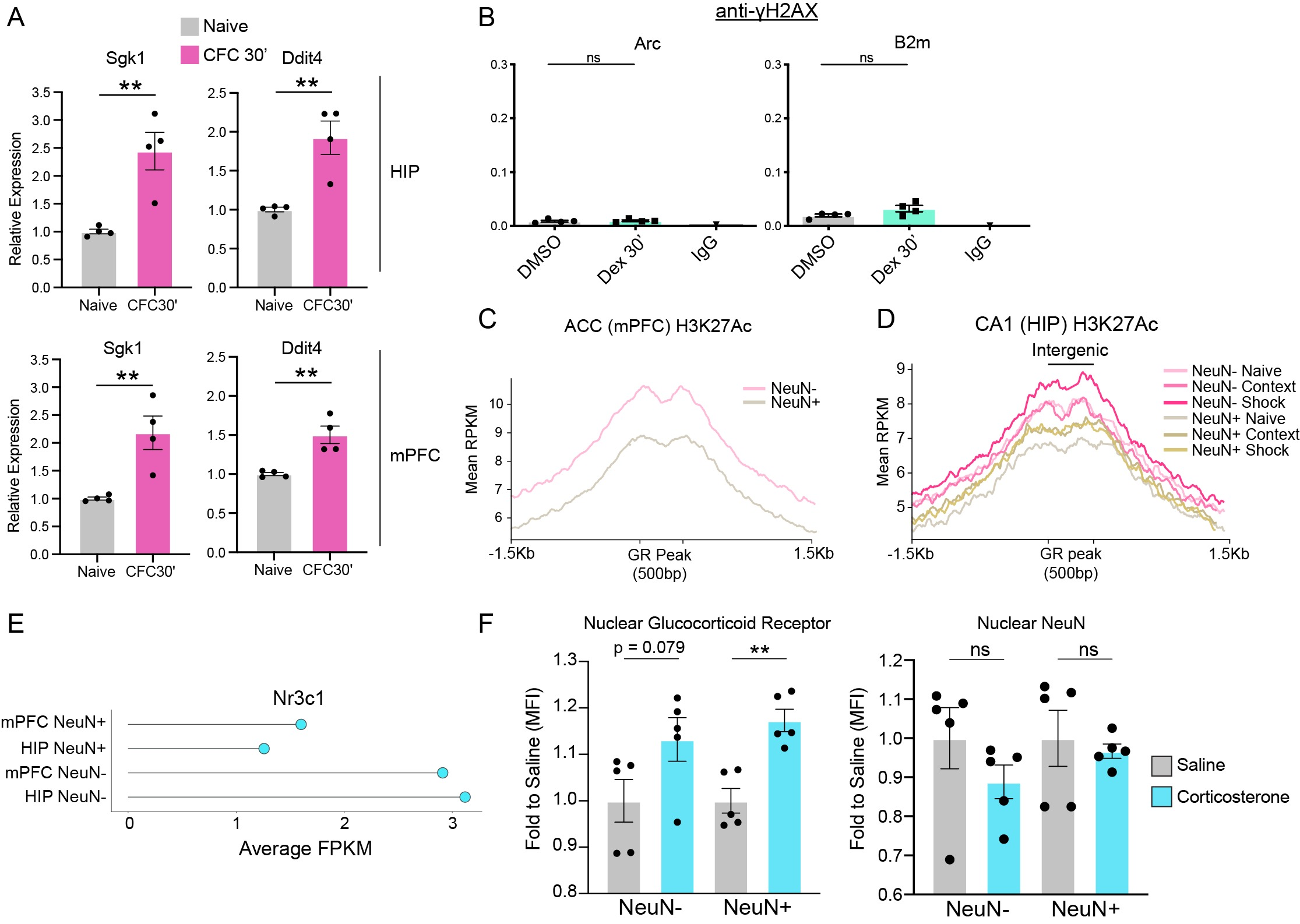

Supplement: S9 Fig — (A) qRT-PCR analysis of Sgk1 and Ddit4 mRNA expression in the HIP and mPFC 30 minutes following contextual fear conditioning—normalized to Hprt and respective naive condition. N = 4 mice per group; two-tailed unpaired student’s t-test; **P ≤ 0.01; Mean ± SEM. (B) ChIP-qPCR analysis of γH2AX induction at select gene bodies in mouse glial primary cultures 30 minutes after treatment with dexamethasone (Dex) (100nM). N = 4 independent cultures; two-tailed unpaired student’s t-test; ns P > 0.05; Mean ± SEM. (C) Average H3K27ac signal of prefrontal anterior cingulate cortex (ACC) at glucocorticoid receptor binding sites (rat cortical ChIP-Seq) [55,56] containing the GC motif in mouse (n = 5591 peaks). H3K27ac ChIP-Seq of naive mouse ACC from NeuN+ or NeuN- isolated nuclei [57]. (D) Average H3K27ac signal at intergenic glucocorticoid receptor binding sites (rat cortical ChIP-Seq) [55,56] containing the GC motif in mouse (n = 1860 peaks). H3K27ac ChIP-Seq of mouse hippocampal CA1 region from NeuN+ or NeuN- isolated nuclei 1 hour after exposure to context, or CFC (shock) [57]. (E) Mean expression of the glucocorticoid receptor (Nr3c1) from naive nuclear RNA-Seq datasets. (F) Intensity of nuclear glucocorticoid receptor (left) or nuclear NeuN (right) in NeuN+ or NeuN- nuclei of the mPFC after corticosterone treatment. Normalized median fluorescence intensity (MFI). N = 5 mice per group; two-tailed unpaired student’s t-test; ** P ≤ 0.01; ns P > 0.05; Mean ± SEM. (TIF) [file pone.0249691.s013.tif]

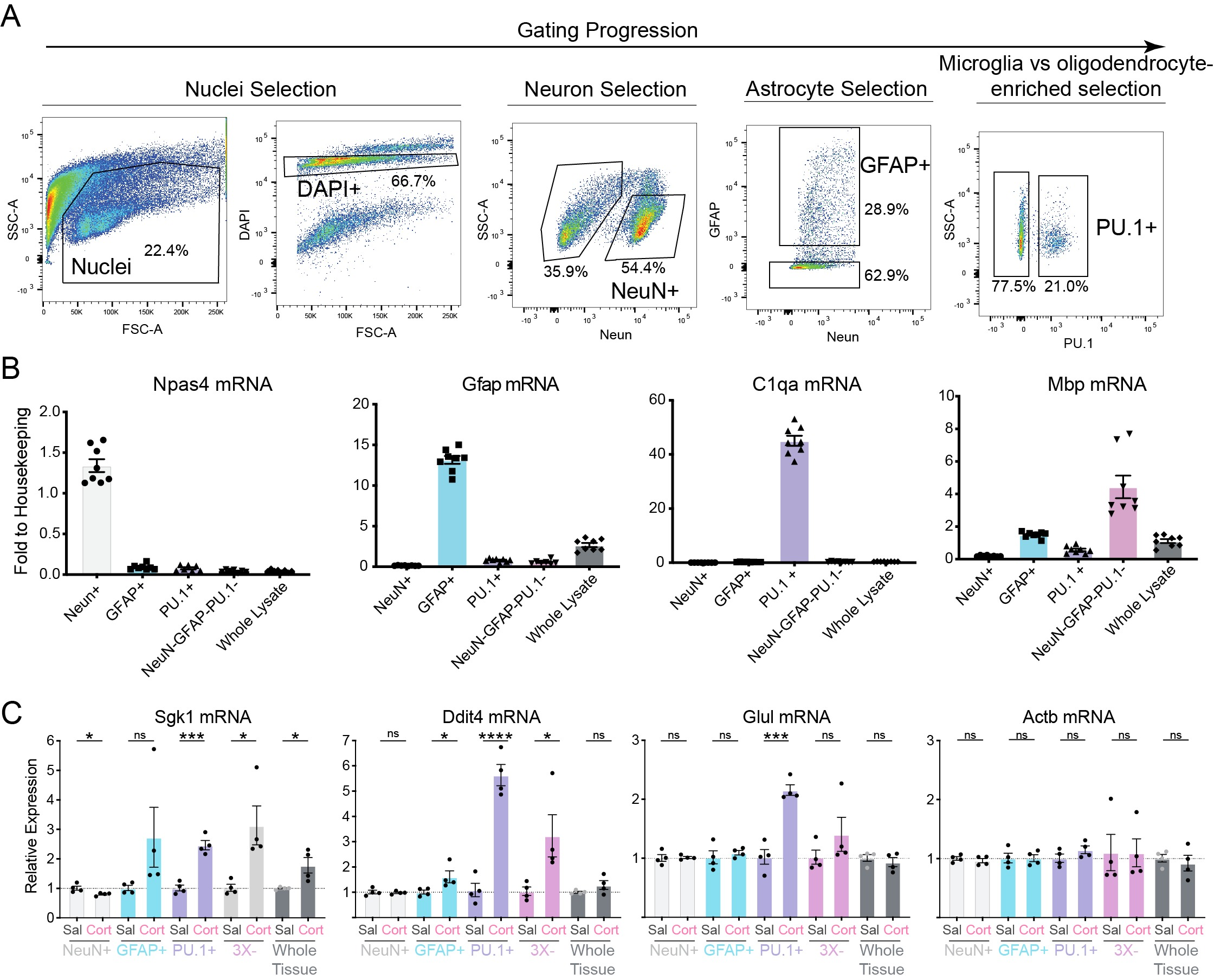

Supplement: S10 Fig — (A) Flow cytometry dot-plots representative of the gating strategy used for isolating neuronal and glial nuclei from hippocampus for RNA-Seq. Appropriately sized (FSC vs SSC plot), singlet nuclei (DAPI+; DNA stain), are gated for the presence or absence of the neuronal nuclei marker NeuN (NeuN+); NeuN- nuclei are then separated by the astrocyte marker GFAP (GFAP+), GFAP- nuclei are then gated for the microglia marker P.1 (PU.1+), with the oligodendrocyte-enriched fraction, NeuN-/GFAP-/PU.1- (3X-), also collected. (B) RT-qPCR analysis of FACS-isolated nuclei for cell-type-specific markers: neuronal (Npas4), astrocyte (Gfap), microglial (C1qa), oligodendrocyte (Mbp). Normalized to Hprt; N = 8, 4 mice saline treated, 4 mice corticosterone treated. (C) Glucocorticoid receptor agonist corticosterone (Cort) induces Sgk1, Ddit4, and Glul in glia, and not the housekeeping gene Actb. RT-qPCR on FACS-purified hippocampal nuclear RNA from neurons (NeuN+), astrocytes (GFAP+), microglia (PU.1+), and oligodendrocytes-enriched (NeuN-GFAP-PU.1-; 3X-) 30 minutes after saline or corticosterone:HBC complex (2mg/Kg) administration. N = 4 mice per group; two-tailed unpaired student’s t-test; * P ≤ 0.05; *** P ≤ 0.001; **** P <0.0001; Mean ± SEM.; * P ≤ 0.05; *** P ≤ 0.001; **** P <0.0001. (TIF) [file pone.0249691.s014.tif]

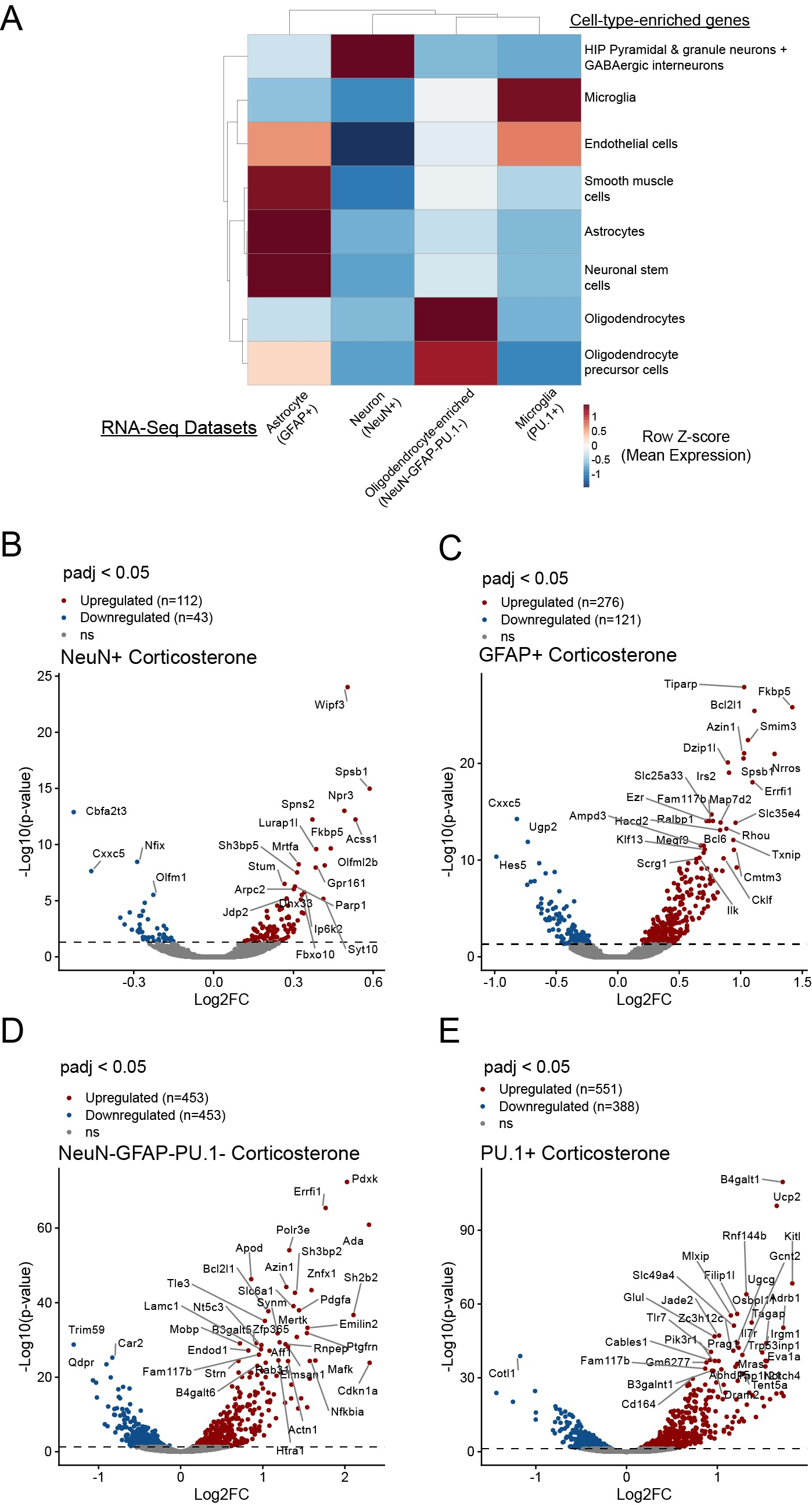

Supplement: S11 Fig — (A) Correspondence between hippocampal cell-type-specific RNA-seq datasets and brain cell types. Marker gene sets for brain cell types was obtained from a previously published dataset, and the average expression of these genes was calculated (RPKM geometric mean) for the saline condition [28]. Z-score determined by row. (B-E) Volcano plots of Log2FC versus Log10(FDR) of RNA-Seq from corticosterone treated hippocampal cell types. Upregulated indicates FDR < 0.05 and log2(FC) > 0, Downregulated indicates FDR < 0.05 and log2(FC) < 0, ns indicates FDR > 0.05. (TIF) [file pone.0249691.s015.tif]

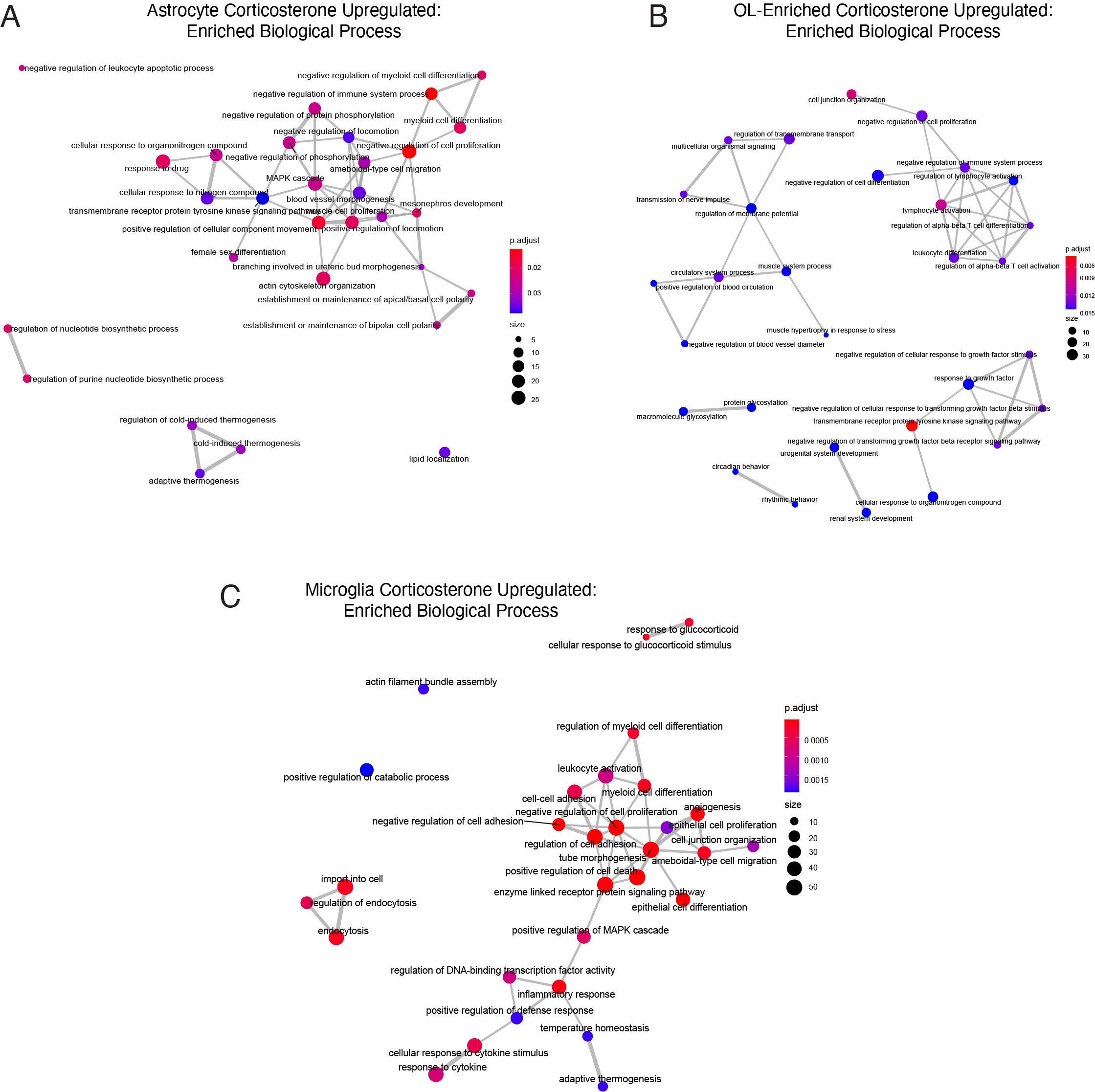

Supplement: S12 Fig — (A-C) Enrichment map of the top 30 enriched biological processes for the genes upregulated in HIP after corticosterone treatment in astrocyte (A), oligodendrocyte-enriched (B), and microglia (C) nuclei. No enrichment in neurons. Over-representation analysis with gene ontology (GO) category “Biological Process.” (TIF) [file pone.0249691.s016.tif]

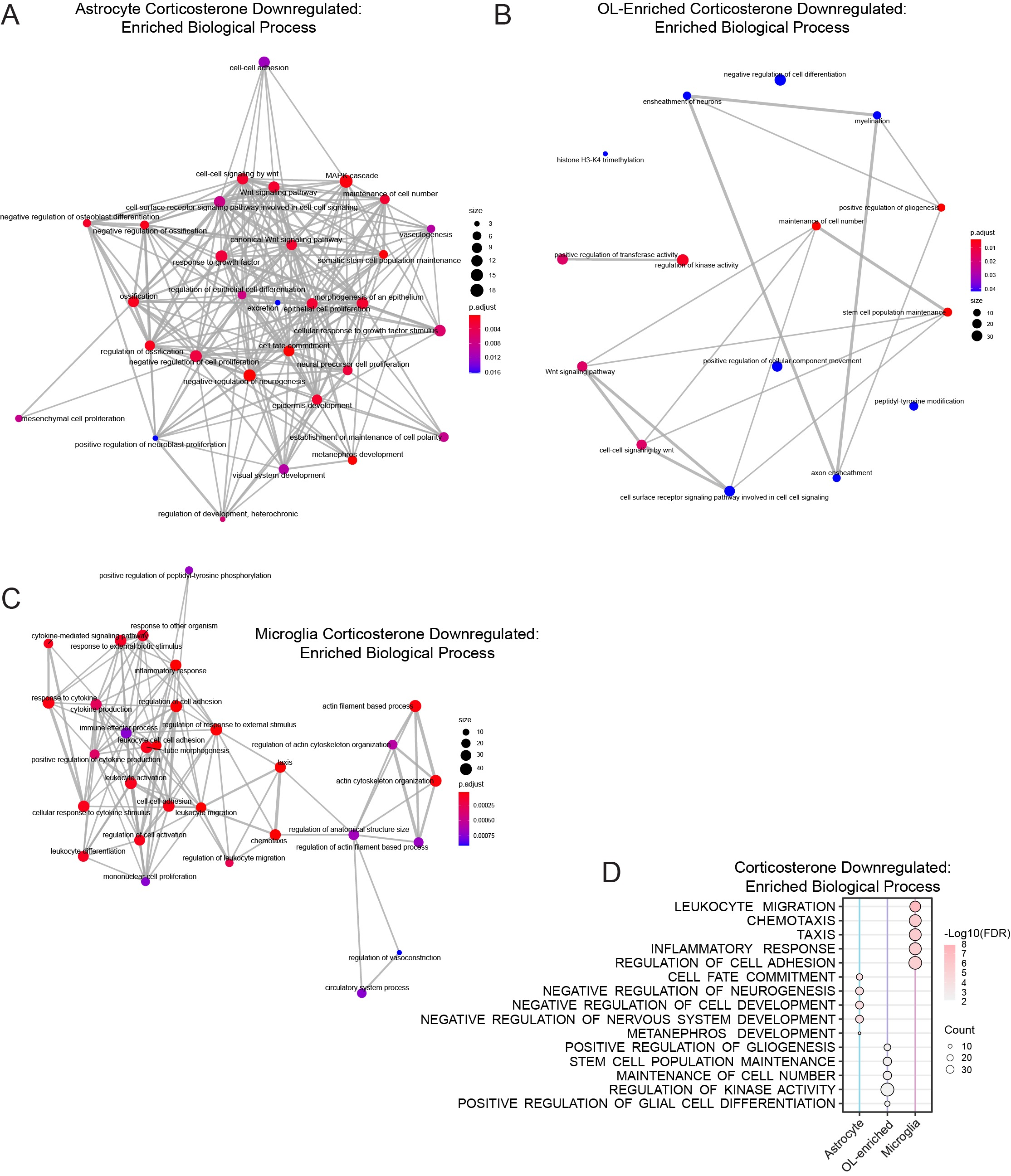

Supplement: S13 Fig — (A-C) Enrichment map of the top enriched biological processes for the genes downregulated in HIP after corticosterone treatment in astrocytic (A), oligodendrocyte-enriched (B), and microglial (C) nuclei. Over-representation analysis with gene ontology (GO) category “Biological Process.” (D) Top five enriched biological processes of the downregulated genes for each cell type in purified hippocampal nuclei from neurons, astrocytes, microglia, and oligodendrocyte-enriched after corticosterone treatment (padj < 0.05). Over-representation analysis with gene ontology (GO) category “Biological Process.” (TIF) [file pone.0249691.s017.tif]

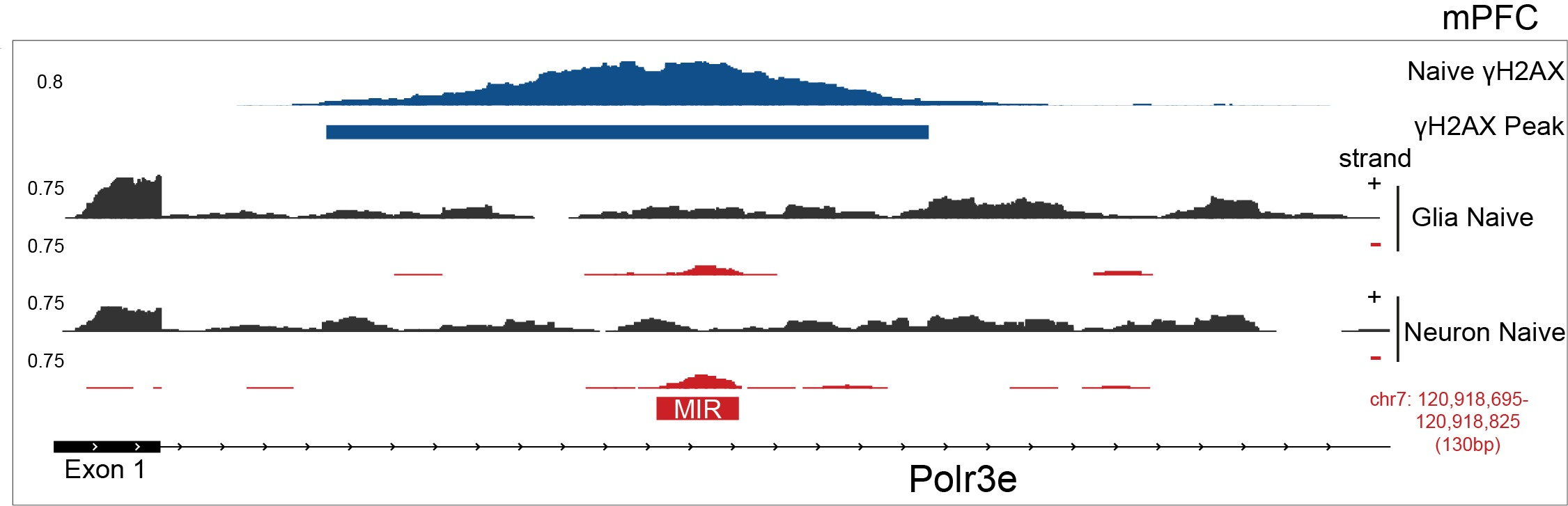

Supplement: S14 Fig — Genome browser tracks for the gene Polr3e displaying a small (951bp) intronic γH2AX peak in mPFC overlapping a mammalian interspersed repeat (MIR). MIR antisense transcription is shown on the negative strand (-). (TIF) [file pone.0249691.s018.tif]

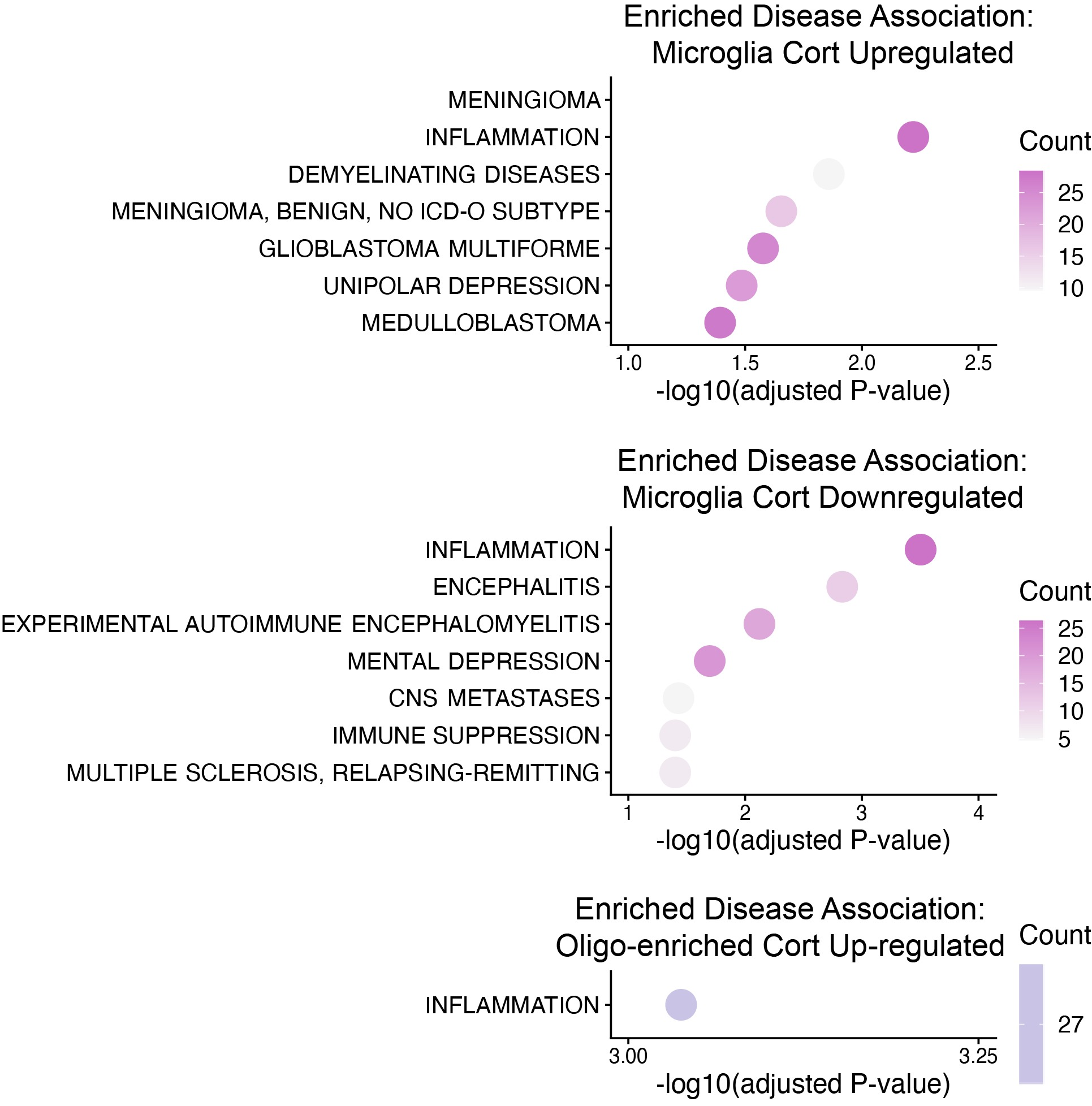

Supplement: S15 Fig — Enriched disease associations for the genes upregulated in HIP after corticosterone treatment in neuronal, astrocytic, microglial, and oligodendrocyte-enriched nuclei, filtered for disease associations related to the nervous system. An absent group indicates no enrichment at threshold q < 0.05. Over-representation analysis with DOSE Disease ontology. (TIF) [file pone.0249691.s019.tif]
