# Supplementary material for: Profiling DNA break sites and transcriptional changes in response to contextual fear learning
Source: PLoS One. 2021 Jul 1;16(7):e0249691. doi: 10.1371/journal.pone.0249691 (PMC8248687; doi:10.1371/journal.pone.0249691)
Supplement: Attachment — Submitted filename: Response to Reviewers.docx [file pone.0249691.s020.docx]

Response to Reviewers

We would like to thank the reviewers for their time and input on this manuscript. We appreciate their comments and suggestions. Please see our responses in red.

Reviewer #1: Unlike cycling cells, where DNA replication and cell cycle progression respond to DSBs and activate the repair mechanism, neuron cells do not proliferate. Hence, it is intriguing how neuron cells handle DSB damage and moreover how DSB formation interplays with the neuronal activities. In this manuscript by Stott et.al, the authors mapped the genome-wide DSB formation in the mouse prefrontal cortex and hippocampus under contextual fear conditioning, which provided an interesting connection between learning behaviors and DNA damage in the brain. I found this research very interesting and technically sound, therefore, recommend its acceptance for publication at PLOS ONE.

We thank the reviewer’s comment.

Reviewer #2: In this study, the authors investigate fear conditioning induced DNA double strand breaks (DSBs) in adult male mouse hippocampus and medial prefrontal cortex through ChIPseq of γH2AX. They recognize widely distributed DSBs after fear conditioning with many demonstrated brain region specific inductions. These DSBs are not only enriched in a few functionally relevant gene categories (e.g. synaptic transmission), but also correlated with transcription changes in neurons and/or glia cells which are profiled through cell-type specific nuclear RNAseq. Furthermore, the upregulated transcripts appear to have higher enrichment of γH2AX and are mediated through activity-dependent gene related transcription factors, which further indicates the functional role of DSBs in neural plasticity. Notably, several fear conditioning induced genes that carry DSBs are modulated by heat shock factor, which suggests an involvement of proteostasis in fear conditioning induced DSBs. Interestingly, many of the γH2AX-containing genes are responsive to glucocorticoid only in non-neuronal nuclei. Following corticosterone administration, the authors find that the differential genes are predominantly in glial cells, with some of them showing high levels of γH2AX. The study includes a large amount of work and the findings are novel. I only have a few minor comments.
1. The study has used γH2AX as a DSB proxy and performed its ChIPseq to identify DSBs genome-wide. It will be helpful to discuss if any limitations of using γH2AX to recognize DSBs. Will DNA single strand break also be detected through this approach?

We agree this is an important point that should be addressed. We have added a new paragraph in the discussion to address the limitations of γH2AX ChIP-seq (please see lines 442-454).

2. Line 82, (B): “six” should be “five”.

We count six for this figure, please see Fig 1B below which is being referenced on line 82:

3. Line 85, (C): Double check if it is top “5” biological processes… or more.

We double-checked and found that we had indeed selected the top five biological processes from each of the four time points.

4. Ling 334: “GC” should be “GR”.

We have corrected this mistake and a few other instances, including in Fig 4.

5. Figure 3A, y-axis: It is unclear why some HSF motifs are shown with a sequence, some are not.

We utilized the given name for each gene set without editing, and some of these names include motifs for reasons unclear. We agree it is a bit confusing, but we did not want to edit them because it could obscure their identity.
